# Human Activity Recognition for Indoor Localization Using Smartphone Inertial Sensors

**DOI:** 10.3390/s21186316

**Published:** 2021-09-21

**Authors:** Dinis Moreira, Marília Barandas, Tiago Rocha, Pedro Alves, Ricardo Santos, Ricardo Leonardo, Pedro Vieira, Hugo Gamboa

**Affiliations:** 1 Associação Fraunhofer Portugal Research, Rua Alfredo Allen 455/461, 4200-135 Porto, Portugal; marilia.barandas@fraunhofer.pt (M.B.); tiago.rocha@fraunhofer.pt (T.R.); pedro.alves@fraunhofer.pt (P.A.); ricardo.santos@fraunhofer.pt (R.S.); ricardo.leonardo@fraunhofer.pt (R.L.); hugo.gamboa@fraunhofer.pt (H.G.); 2 Laboratório de Instrumentação, Engenharia Biomédica e Física da Radiação (LIBPhys-UNL), Departamento de Física, Faculdade de Ciências e Tecnologia, Universidade Nova de Lisboa, 2829-516 Caparica, Portugal; 3 Instituto de Telecomunicações, Instituto Superior Técnico, 1049-001 Lisboa, Portugal; pedro.vieira@isel.pt; 4 Departamento de Engenharia Electrónica e Telecomunicações e de Computadores (DEETC), Instituto Superior de Engenharia de Lisboa, 1959-007 Lisboa, Portugal

**Keywords:** smartphone, inertial sensors, deep learning, human activity recognition, indoor location

## Abstract

With the fast increase in the demand for location-based services and the proliferation of smartphones, the topic of indoor localization is attracting great interest. In indoor environments, users’ performed activities carry useful semantic information. These activities can then be used by indoor localization systems to confirm users’ current relative locations in a building. In this paper, we propose a deep-learning model based on a Convolutional Long Short-Term Memory (ConvLSTM) network to classify human activities within the indoor localization scenario using smartphone inertial sensor data. Results show that the proposed human activity recognition (HAR) model accurately identifies nine types of activities: not moving, walking, running, going up in an elevator, going down in an elevator, walking upstairs, walking downstairs, or going up and down a ramp. Moreover, predicted human activities were integrated within an existing indoor positioning system and evaluated in a multi-story building across several testing routes, with an average positioning error of 2.4 m. The results show that the inclusion of human activity information can reduce the overall localization error of the system and actively contribute to the better identification of floor transitions within a building. The conducted experiments demonstrated promising results and verified the effectiveness of using human activity-related information for indoor localization.

## 1. Introduction

Human activities have been commonly used to define a vast number of human behavioral patterns. A human activity can be defined as a set of actions that can be repeated over time in a given environment [1,2]. By monitoring human activity, it is possible to extract knowledge from a user and their environment for a variety of applications. With the increasing number and availability of sensors embedded in mobile devices, it has become possible to develop distinct practical applications in several different areas of research [1,2,3,4]. Fall detection systems, ambient assisted-living applications for elderly monitoring, or disease prevention solutions are some representative examples within the healthcare field [1,2,5]. Additionally, HAR has also been used to recognize individual activities in different areas, such as Internet of Things and Smart Cities, security, localization, and transportation [1,3]. Generally, HAR systems aim to determine the ongoing actions/activities performed by a person or group of persons based on sensory observation data to extract knowledge of the context where the activity took place [1,2,4,6].

The literature on this topic is quite vast and is frequently grouped depending on the relevant problem, type of available sensors, and the total number and complexity of monitored activities [1,2,3,4]. Most HAR solutions have been developed using several machine learning (ML) methods, including both classic ML and deep-learning (DL) algorithms. Generally, some of the classic ML algorithms use Support Vector Machines (SVM), Decision Trees, Naive Bayes, and K-Nearest Neighbors (KNN). Additionally, deep-learning (DL) algorithms use Convolutional Neural Networks (CNN), Recurrent Neural Networks (RNN), Restricted Boltzmann Machines (RBM), or Stacked Autoencoders (SAE) [1]. The main difference between these two approaches is that traditional methods require a manual computation of the features to be fed to the classifier, which may be limited by the user’s domain knowledge, while DL methods typically have the feature extraction step embedded in the network, which automatically captures and calculates these features [1,4]. Thus, there is a clear shift from more traditional ML methods to more advanced approaches, usually related to DL.

Specifically, one of the areas that may greatly benefit from HAR is the indoor localization field, where the standard Global Positioning System (GPS) is usually not available. As such, HAR may play a key role in extracting additional context information about the underlying activities that are being performed by users indoors, acting as an additional and auxiliary layer of information for any Pedestrian Dead Reckoning (PDR) system [4,7,8]. For example, the detection of human activities such as “moving upstairs or downstairs” or “taking an elevator” may help to localize a user in this scenario, reducing the localization uncertainty within a building [4,7].

Traditionally, Indoor Positioning Systems (IPS) resort to different layers of information to improve the localization performance. Infrastructure-based solutions rely on equipment installed throughout buildings, such as Bluetooth beacons, which communicate with the moving devices to produce a location estimation given the floor plan [9]. On the other hand, infrastructure-free solutions aim at reducing the implementation and maintenance costs by resorting to pervasive sources of information, such as the magnetic field or ubiquitous Wi-Fi networks [10]. The different information sources are prone to noise and interferences, harming the positioning results in most systems. To tackle these limitations, more layers of information are being leveraged, from which the HAR emerges as an important contribution to provide context information. Using the inertial sensors from the moving devices, IPS can understand the movement characteristics, either when walking throughout a plane or when crossing floors. With these accurate estimations, multi-layer systems can balance contradicting erroneous information from other sources, therefore providing more accurate positioning.

In this paper, we propose a deep-learning-based method for HAR in indoor environments using a combination of inertial data from smartphones’ built-in sensors. A Convolutional Long Short-Term Memory (ConvLSTM)-based deep neural network [11] is proposed to automatically capture time-related dependencies and extract spatial features for the identification of nine different human activities, including not moving, walking, running, moving upstairs, moving downstairs, going up in an elevator, going down in an elevator, or walking up or down a ramp.

The major contributions of this paper are the demonstration of effectiveness of the following:

- A deep-learning framework that relies only on inertial sensors to accurately recognize nine different activities;
- The training of the HAR model using data from a vast number of different users and several different low- to high-range smartphones, increasing the model’s robustness;
- Using a human activity recognition module within an indoor positioning system, improving the overall positioning results.

The remainder of this paper is organized as follows. The related works are briefly reviewed in Section 2. Section 3 provides a background overview of convolutional and recurrent neural networks. Section 4 presents the proposed methodology. Section 5 presents the experimental results. The performance analysis and discussion of results are presented in Section 6. Finally, the conclusions and future recommendations are presented in Section 7.

## 2. Related Work

During the last decade, significant research work has been conducted within the HAR field to better understand and identify human behavioral patterns for different applications. Ambient assisted living, healthcare, Internet of Things, security, location, and navigation-related solutions were developed in recent years using contextual information extracted by HAR modules [12,13]. HAR can be performed with a variety of sensors and devices [14]. Smartphones have become the gold standard for performing this type of tasks due to their reduced cost, ubiquitous usage, and the capabilities of their sensors both to capture human movement and “feel” the surrounding environment. Currently, almost every person has access to a smartphone, which, combined with the fact that it is a mobile device that can be carried around, makes smartphones the ideal candidates for performing HAR classification [1]. For the purpose of this study, we only considered, for comparison, mobile-related solutions that used smartphones’ embedded sensor data.

Despite HAR being an already explored topic, it is still quite far from reaching its full potential. Most studies have only aimed at classifying simpler and traditional activities, such as sitting, standing, laying, walking, running, and going up or down the stairs [1,13,14,15]. Within this scenario, the number of publications in the literature is quite vast and has demonstrated promising results (with an average accuracy over 90%) [1,13,14,15]. However, as the number and complexity of the activities for classification increase, the volume of publications decreases, making it harder to accurately compare results among reported studies. These newer and more complex activities may depend on the purpose of each HAR system and subsequently be more related to a specific domain. For instance, the higher complexity can be associated with specific activities of daily living, such as detecting the opening of a door or the action of brushing teeth, or they could be related to an indoor navigation scenario, such as detecting a user when using an elevator or when going up or down a ramp. Thus, and for the purpose of this study, we only consider solutions that target the latter scenario. Moreover, exploring human activities in this sense may increase the capability of a given system to infer a user’s location more precisely and provide means for navigation within a building.

### 2.1. Traditional Machine Learning Methods for HAR

Traditional ML methods were often considered and employed to solve almost all classification tasks. Usually, they consist of two main parts: the extraction of meaningful/discriminant features and classification. The feature extraction process requires human intervention for the design and computation of features and usually depends on the user’s domain knowledge. Time domain features, frequency domain, and others (custom features) were widely used for this purpose [4,16]. For the classification task, a variety of classic methods, such as Decision Trees, SVM, KNN, Markov models, or regression methods, were widely used and reported in the literature [1].

### 2.2. Deep-Learning Methods for HAR

In the past few years, DL techniques have been demonstrated to be successful when applied in a variety of domains, usually surpassing previous state-of-the-art results. Moreover, as these methods can be modeled to automatically perform the feature extraction step, together with their overall superiority in many tasks and in terms of classification results, DL-based techniques have quickly attracted considerable attention [1,5,6]. Specifically for HAR, several DL architectures have been proposed in the literature with quite promising results.

Several convolution-based neural networks that are widely used in the image processing domain were proposed for the classification of several human activities [4,5]. Zhou et al. [4] applied a CNN using accelerometer, gyroscope, magnetometer, and barometer data from 10 different subjects for the classification of nine different activities: walking, standing, turning, walking up stairs, going up or down an escalator, and going up or down in an elevator, with an overall accuracy of 98%. In the work of Murad et al. [17], a deep recurrent network architecture based on LSTM was proposed for the classification of six different activities of daily living (ADL), namely walking up/downstairs, sitting, standing, and laying. This network attained an overall accuracy of 96.7% and 97.8% in the UCI-HAD and USC-HAD datasets, respectively, by using three-axial accelerometer, gyroscope, and magnetometer data from a smartphone. Several other DL methods have been reported in the literature that were already applied in the context of HAR [1,8], ranging from deeply connected networks (DFN), RBM, and SAE to custom combinations of CNN, RNN, and LSTM [8]. However, it is quite difficult to compare all the related works in this area as they may differ in the types of classified activities, the used sensors, and/or the specific context in which they were applied (e.g., ADL vs indoor positioning scenarios).

### 2.3. Indoor Localization with HAR

Indoor localization has been an intense research topic in recent years. Although infrastructure-based solutions depend on dedicated equipment, which increases deployment costs but produces more accurate estimations, infrastructure-free systems leverage pervasive and ubiquitous information, at the cost of reducing the localization precision. In this sense, HAR, used as an additional layer of information, can help IPS to provide accurate positioning.

Most solutions limit HAR to the identification of movement, followed by step detection and heading and stride estimation, to reconstruct users’ trajectories. As such, PDR is used in the work of Guimarães et al. [10] together with a particle filter and more layers of information to provide continuous location estimation. Additionally, Guo et al. [7] developed an IPS that leverages HAR using inertial sensors and a barometer to detect specific activities, such as going up stairs or opening doors, which are then matched using previously annotated landmarks in the floor plan, aiding improvements to the positioning process. Using sound as a source for HAR, Galván-Tejada et al. [18] developed a room-level IPS, leveraging a Random Forest model to identify the specific features of activities traditionally performed in the same location.

With another objective, Zhou et al. [19] developed an algorithm for the deployment stage of IPS, leveraging HAR to cluster specific activities performed in different areas of the buildings, which are then used in the automatic construction of the floor plan. From a similar perspective, Wang et al. [20] detected specific activities, such as using the elevator or the stairs, to aid in the detection of the corresponding areas on the floor plans.

## 3. Background: Neural Networks

### 3.1. Convolution Neural Networks

**Figure 1:**
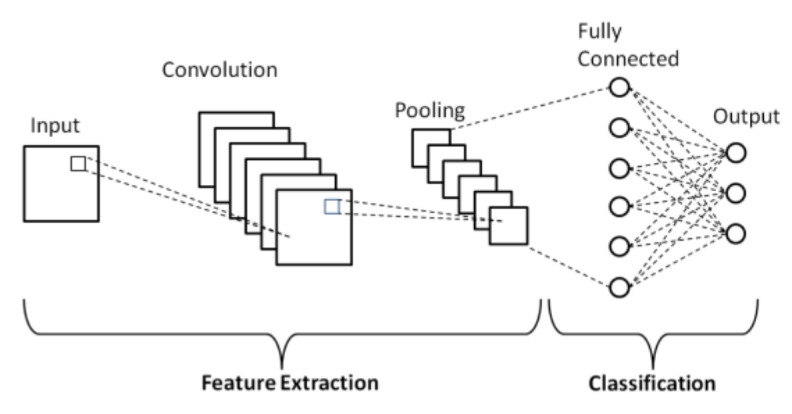
Schematic diagram of a basic CNN architecture [24].

A CNN is usually composed of two parts: convolution and pooling operations. These operations are applied consecutively and act as a deep feature extractor of the raw data. Additionally, these features are connected to a fully connected layer that produces the output classification (see Figure 1). The convolutional layer performs convolution operations on the data of the preceding layer with a given convolution filter and activation function, generating a set of different feature maps. The total number of filters, filter/kernel size, convolution stride, and padding are just some of the parameters that can be tuned and modified according to the problem at hand. The pooling layer performs a downsampling operation with a given kernel/size that reduces the size of the generated feature maps by representing its values by its average or maximum value according to the kernel [21,22,23].

One benefit of using this type of networks is that a single weight matrix is learned in each layer to generate the different feature maps, and each neuron is only connected to a subset of the input, as opposed to regular neural networks, thus reducing the total number of learnable parameters of the model, which makes the train faster and more efficient [21,22,23]. CNNs have become extremely popular for a variety of tasks, mostly when applied to images, due to their basic structure and capability of extracting contextual spatial features from inputs [23].

### 3.2. Long Short-Term Neural Networks

The RNN is a cyclic network that is widely used to model sequential data. As opposed to traditional neural networks, RNNs do not assume that all inputs are independent of each other and use a looping mechanism to retain some “memory” about past elements of the input sequence. Traditional RNNs suffer from the short-term memory and vanishing/exploding gradient problems, meaning that they are not suitable for remembering long-term dependencies in the past, especially for arbitrarily long sequences, and may stop learning early in the process if the error’s gradient value during the backpropagation is too small or too big [17]. Thus, in order to solve this issue, a more complex type of RNN, called LSTM, was created.

**Figure 2:**
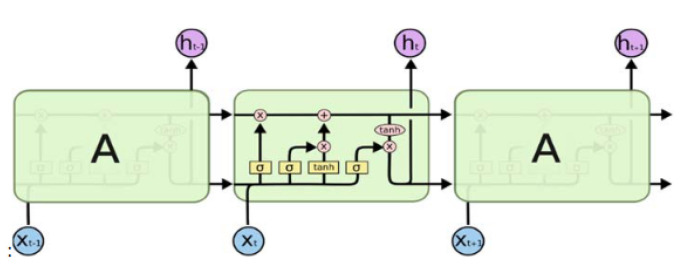
Schematic diagram of a basic LSTM block [25].

LSTM, instead of using nodes, is composed of special “memory blocks” containing three key gate units: a forget gate, input gate, and output gate (see Figure 2). These gates essentially control the amount of information that the network should keep and forget from the original input sequence. Concretely, the forget gate decides which information should be thrown away from the cell state, the input gate decides which new information is added to the cell state, and the output gate decides how much of the internal state should be passed to the next step. By using this gating mechanism, LSTMs can explicitly model long-term dependencies, making them attractive for a variety of time-related problems [17].

### 3.3. Convolution LSTM Neural Networks

It is widely known that every model architecture comes with its own advantages and disadvantages. CNNs are known and used for the extraction of spatial features from input data; however, they fail when it comes to learning temporal relationships present in the data. On the other hand, LSTMs are quite effective at or capable of modeling long-term dependencies and are therefore excellent for modeling sequences; however, they can be ineffective at extracting spatial information from the inputs. Thus, and to better model this spatio-temporal relationship in the input data, a new type of network that combines the advantages of the two aforementioned networks was first introduced by Shi et al.: Convolution LSTM (ConvLSTM) [11].

**Figure 3:**
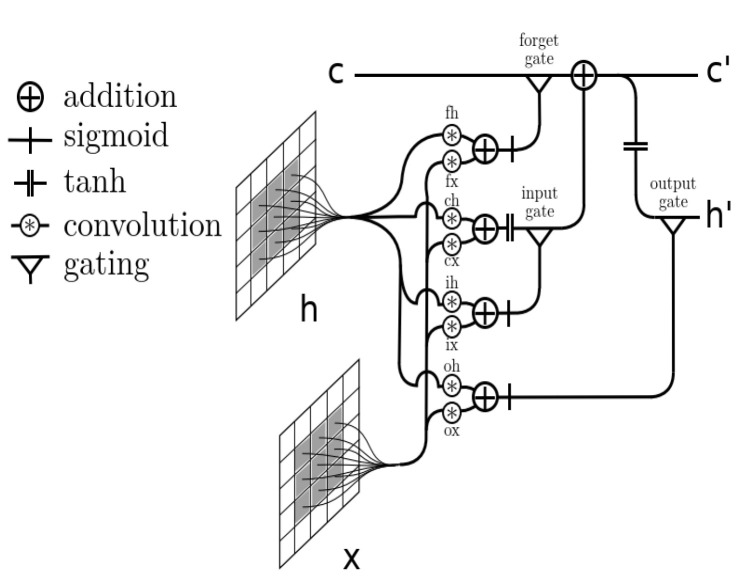
Schematic diagram of a ConvLSTM layer [26].

ConvLSTM is based on an LSTM architecture where the internal matrix multiplications are exchanged by convolution operations (see Figure 3). These convolution structures are applied both at the input-to-state transition and at the state-to-state transitions and are able not only to capture the spatial context information present in the input data, as a typical CNN, but also to model the long-range dependencies within the time sequences, as a typical LSTM.

## 4. Methodology

**Figure 4:**
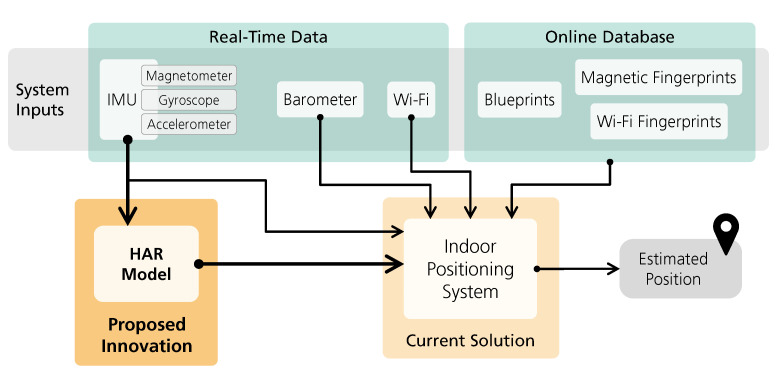
Overview of HAR model integration in an indoor positioning system.

The adopted methodology for the integration of the HAR model in an indoor positioning system is presented in Figure 4. First, a deep-learning framework that relies only on inertial sensors was trained to recognize nine human activities: not moving, walking, running, riding an elevator up, riding an elevator down, walking upstairs, walking downstairs, or going up and down a ramp. These activities were selected due to their potential to provide context information for indoor positioning and navigation. After training and validation, the proposed HAR model was integrated in a fingerprinting-based indoor positioning system based on the work of Guimarães et al. [10] and on the improvements of step detection and particle filter algorithms from the work of Santos et al. [27].

In the following sections, a detailed description of the proposed HAR model is presented, followed by a description of the indoor positioning system used.

### 4.1. Proposed HAR Architecture

**Figure 5:**
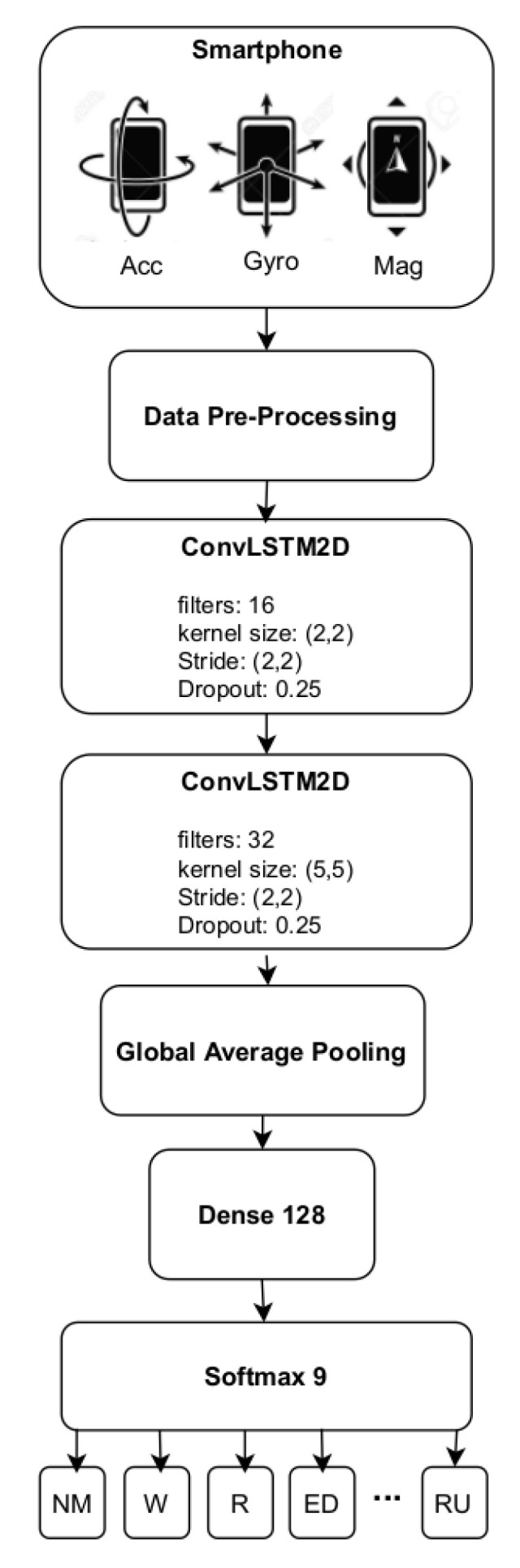
Proposed HAR architecture.

A schematic diagram that describes the architecture of the proposed HAR system is presented in Figure 5. The proposed architecture consists of two stacked ConvLSTM layers (dropout: 0.25) with 16 and 32 filters, followed by a pooling layer (Global Average Pooling) and a fully connected layer before the final softmax layer.

The proposed system performs the direct end-to-end mapping of raw inertial sensor inputs—namely three-axis accelerometer, gyroscope, and magnetometer values—together with their correspondent magnitude values to an activity classification label. Thus, the input is a discrete sequence of equally spaced samples $\left(x_{1},x_{2},\ldots ,x_{T}\right)$, where each data point $x_{t}$ is a vector of individual samples collected by each sensor at time *t*. These samples are segmented into windows of fixed length, representing a given time window *T*, which are then fed to the model. The model outputs a sequence of probabilities, representing the activity label predictions for a given time window.

#### 4.1.1. Data Pre-Processing

As pre-processing methods, data resampling, filtering, segmentation, and scaling were employed. In the following sections, descriptions of these techniques are presented.

##### Resampling

To deal with the sensor’s different sampling frequencies and to define a fixed temporal resolution, a data-resampling strategy was employed. Data from each of the sensors were resampled to obtain a sampling frequency of 50 Hz. Moreover, acquiring sensor data at this rate has been found to be sufficient for capturing human body motion as 99% of its energy is contained below 15 Hz [28].

##### Filtering

Generally, concerning time series, it is quite frequent to use some strategy for noise reduction. The measured values from any given sensor are subject to some uncertainty, which can be significantly higher if noise is present in the signal. Moreover, performing complex activities with a variety of consecutive movements and the continuous streaming of inertial sensors data recorded over time may increase the presence of noise in the recordings. Thus, a sliding median filtering technique was applied to the input data. A window of nine points was used to slide over each sensor sequence of values to replace its central value by the current window median value. This operation was able to create a smoother version of the original signal, reducing the presence of outlier values in those sequences that could compromise the model’s ability to learn during training.

##### Segmentation

The recorded data correspond to a time-varying signal that needs to be discretized into fixed-length training examples, with each example being an activity in this case. Thus, an overlapping sliding window approach was implemented for data stream segmentation. Since the selection of the window size may be considered an empirical and task-oriented problem—i.e., closely related to the duration of the event(s) of interest—based on a literature review, a window size of 2.5 s with a fixed overlap of 0.5 s was empirically chosen. Several studies show that the ideal window size for human activity recognition varies around 2 to 5 s considering a sampling frequency of 20 to 50 Hz [1,4].

##### Scaling

Since data from three different sensors were used, represented in different scales, a scaling technique was added to the pipeline. The purpose of performing this scaling step was to standardize the independent feature values present in the data to the same fixed range. This helped in the handling of highly varying magnitudes, values, or units present in the input data [29]. Moreover, when using gradient descent optimization, the presence of a higher feature value in the sample affects how the step size of the gradient descent changes, causing different step sizes for each feature, which may slow the gradient convergence during training [29]. Thus, a standardization technique based on the mean and standard deviation of the features values was applied to re-scale each independent feature value so that it had a distribution with zero mean and unit variance.

#### 4.1.2. Model Training and Optimization

The proposed HAR model was trained using a fixed set of data (training dataset) and evaluated using an independent set of data (test dataset). Test data were collected in new locations and buildings that were never seen during the training phase and thus were ideal for performing a robust analysis of the model performance. All the model weights (parameters) were initialized using the Glorot uniform initialization technique and updated at the end of each epoch to minimize the loss function. The ground truth labels for each segmented window were provided as one-hot-encoded vectors, and the categorical cross entropy loss was selected as the loss function. The Adam optimization algorithm with a learning rate of 0.001 was used to backpropagate the error gradient and update the model’s weights. The dropout technique was used during training to avoid overfitting, with a dropout value of 0.25 selected for the first two ConvLSTM layers.

The training data were fed to the model using a custom data generator that allowed data shuffling after the end of each epoch and enabled the control of the label distribution within each batch of data, ensuring the generation of balanced batches in terms of different activities. The goal of using this custom data generator was to mitigate the fact that we were faced with an extremely unbalanced dataset. This ensured that the model could always see some examples of each activity during the training of each mini-batch. Moreover, during the data generation process, and for each segmented window, the ground truth label was defined according to the activity most represented in that given time interval, based on the existing ground truth annotations. It is worth mentioning that a sample weighting strategy was also used for model training.

The correspondent sample weight for each generated window was defined as equal to the correspondent time fraction that the most represented activity has within the given time window (maximum sample weight: 1.0). On one hand, if only a single activity was present during the whole window duration, its label would be the exact same activity with a sample weight of 1.0. On the other hand, if a given window contained more than one activity within the window duration—for example, the user was walking and running 30% and 70% of the time, respectively—that window would be labelled as running with a sample weight of 0.7. Thus, smaller sample weights were attributed to time windows that contained transitions between different activities and higher weights to time windows that contained information of only a single activity. The model learning process benefitted from this approach due to the smaller loss obtained when a misclassification occurred on a window with a transition between activities. In contrast, a greater loss was obtained when a misclassification occurred on a window containing only a single activity. Thus, by using this approach, we increased the variability of the training data by presenting the kinds of boundary situations that occur in practice without compromising the training process and by forcing the model to learn how to deal with these cases.

### 4.2. Indoor Positioning System

For the integration of the proposed HAR model, a fingerprinting-based indoor positioning system based on the work of Guimarães et al. [10] was used. The solution uses an intelligent fused positioning mechanism based on a particle filter implementation to opportunistically join multiple sources of information, according to the knowledge of the system state, available observations, and fingerprints. In the next subsections, the human motion tracking and the particle filter details are briefly introduced.

#### 4.2.1. Human Motion Tracking

Human locomotion is characterized by the generation of movement (i.e., steps) to progress in a direction. A smartphone, carried by its user, is capable of sensing three-dimensional inertial and environmental data.

Data from the accelerometer, magnetometer, and gyroscope were combined using a second-order complementary filter to compute the orientation of the device relative to the Earth frame with increased accuracy, as in [10]. Regarding PDR techniques, the implementation of step detection, step length, and heading variation was based on the works of Guimarães et al. [10] and Santos et al. [27].

Step detection is achieved using a CNN. The network takes as its input a sliding window of 1.28 s of acceleration data transposed to the reference frame of the user, using the method proposed in [30]. The output is a time series and can be interpreted as the probability of the presence of a step at each instant. A detailed description of the step detection algorithm is presented in [27].

The length of each step (*l*) is computed using the method proposed by Weinberg [31]:

(1) $$l=K\sqrt[{4}]{A_{max}-A_{min}}$$

where $A_{max}$ and $A_{min}$ are the maximum and minimum values of the vertical acceleration in the Earth reference frame for that step and *K* is a calibration constant, adjusted using a recursive least-squares method and used for unit conversion (i.e., meters travelled). *K* was empirically calibrated for a typical person and was set to 0.45.

Finally, the heading variation at each step was computed through the numerical integration of the *z* component of the gyroscope in the Earth’s reference frame. With all the obtained movement parameters, it was possible to estimate consecutive positions between steps.

#### 4.2.2. Particle Filtering

Particle filtering is a well-known technique that enables a system to estimate recursively the position of the user from a set of measurements. Essentially, a particle filter is based on a set of random samples with weights, called particles *p*, representing the possible positions and directions of a user. A particle is represented by (x,y,z,h,s) attributes, where (x,y) is a 2D pair coordinate, *z* represents the floor number of a building, *h* is the particle heading, and *s* is the particle significance. With the exception of the system’s initialization, the generation of particles is conducted each time the motion model predicts a step. Based on the motion model measurements (i.e., step length and heading variation) and on the observations from the environment (i.e., architectural, magnetic, and Wi-Fi information), the particles’ significance is updated.

Regarding the initial localization process, the system probes for Wi-Fi information to estimate the current location area by matching the Wi-Fi fingerprints. The received Wi-Fi events at the target place are matched to the Wi-Fi fingerprints based on their Basic Service Set Identifier (BSSID) and radio band, resulting in a set of possible positions. The result of this process is a set of clusters containing multiple position and heading hypotheses, which are used as starting particles. The significance of each possible particle ($p_{s}$) is calculated using the geometric mean of the Gaussian distance between the measured signal strength of an access point *j* ($s_{f}^{j}$) and the corresponding fingerprint value ($s_{m}^{j}$) at that position by Equation (2):

(2) $$p_{s}={\left[\prod_{j=1}^{n}exp\left[\frac{1}{2}{\left(\frac{s_{m}^{j}-s_{f}^{j}}{\sigma_{w}}\right)}^{2}\right]\right]}^{\frac{1}{n}}$$

where *n* is the number of visible access points registered in the Wi-Fi fingerprint and $\sigma_{w}$ is the standard deviation of the Gaussian distribution, here set to 5 dB.

Then, as the user moves, environmental and motion information is collected and matched against the fingerprints and the architectural building information, such as walls, corridors, elevators, and stairs, which iteratively removes erroneous hypotheses until only the most accurate ones remain.

At each detected step, a step length $l^{\prime }$ and a heading variation $d\theta^{\prime }$ are sampled for each particle from Gaussian distributions *L* and dΘ as follows:

(3) $$L∼N(l,l\times \sigma_{l}^{2})$$

(4) $$d\Theta ∼N(d\theta ,\sigma_{\theta }^{2})$$

where *l* is the length of the step as determined by Equation (1) and dθ is the original difference between the heading of the current step and the heading of the previous step. $\sigma_{l}^{2}$ and $\sigma_{\theta }^{2}$ refer to the variance of the *L* and dΘ distributions, respectively. These are applied to the position of the particle as follows:

(5) $$\begin{array}{cc} \; & p_{h}^{i,t}=p_{h}^{i,t-1}+d\theta^{\prime }\\ \; & p_{x}^{i,t}=p_{x}^{i,t-1}+l^{\prime }\cos\left(p_{h}^{i,t}\right)\\ \; & p_{y}^{i,t}=p_{y}^{i,t-1}+l^{\prime }\sin\left(p_{h}^{i,t}\right)\\ \; & p_{s}^{i,t}={\left(p_{s}^{i,t-1}\right)}^{1-w_{o}}\times {\left(p_{o}^{i,t}\right)}^{w_{o}} \end{array}$$

where $p^{i,t}$ refers to particle *i* at step *t*, characterized by its heading *h*, 2D position (x,y), and significance *s*. $p_{o}$ is the likelihood of observation *o*, and $w_{o}$ is the weight of observation *o* used for the geometric combination with the previous particle significance. The geometric mean of likelihoods enables the system to trust differently for each considered layer and enables the inclusion of other reference features besides those currently explored. The observations included in this study were the dead reckoning measures, magnetic field, Wi-Fi, human activities, and the architectural building information.

The particle filter was implemented so that the lack of a given observation would not compromise the ability of reporting positions. Therefore, when an observation was available, it was combined with the current system state.

Due to the high sampling frequency of magnetic field signals in common smartphones (approximately between 50 Hz and 200 Hz), the likelihood of the magnetic field was combined with the likelihood of dead reckoning measures. Therefore, the likelihood of the motion tracking layer ($p_{o=mov}^{i,t}$) is given by Equation (6).

(6) $$\begin{array}{c} p_{o=mov}^{i,t}=\frac{\left[P\left(L=l^{\prime }\right)\times P\left(d\Theta =d\theta^{\prime }\right)\right]\times w_{dr}+P_{mag}\times w_{mag}}{w_{dr}+w_{mag}} \end{array}$$

where $w_{dr}$ and $w_{mag}$ are the weights for the combination of dead reckoning and the magnetic field, respectively. $P_{mag}$ is the likelihood of the magnetic field calculated using a Gaussian distance between the measured magnetic values (each time a step is detected) and those stored in the fingerprint.

Regarding the likelihood of the Wi-Fi layer ($p_{o=wifi}^{i,t}$), it is equal to the particle’s significance in the system initialization process, given by Equation (2).

For the human activities layer, $p_{o=har}^{i,t}$ uses the architectural building information together with the outputted HAR activities probabilities to increase/decrease each particle significance given its particular position on the map, accordingly. For instance, in a particular time instant and if the model predicts an upstairs activity, every particle in the stairs positions will see its significance increased, according to the model’s outputted upstairs probability, while the particles in walkable areas or elevators will decrease their significance. The latter feature would be particularly important to recognize and deal with floor transitions within the building, either by stairs, elevators or ramps, in a better way. By crossing activity and architectural building information, the uncertainty of a user’s relative location in the building can be reduced.

To counteract sample redundancy, particles have their significance ($p_{s}$) penalized when occupying the same grid square as other particles. This is achieved by applying the following Equation:

(7) $$p_{s}^{i,t}=\frac{p_{s}^{i,t}}{C^{0.5\exp(-0.5\frac{N}{A})}}$$

where *C* is the number of particles in the same grid square as $p^{i,t}$, *A* is the total number of occupied grid squares, and *N* is the maximum number of particles.

Finally, the particles are resampled with replacement. The probability of each particle being sampled is proportional to its significance.

## 5. Experiments and Results

### 5.1. Datasets

To train and evaluate the proposed model, two different datasets were considered: (1) an HAR dataset containing diverse motion data recorded by volunteers performing nine activities, and (2) an indoor positioning dataset containing multiple floor transitions for assessing HAR model integration within the IPS scenario. In the following sections, a description of the considered HAR activities is presented, followed by a description of both datasets.

#### 5.1.1. HAR Activities

In indoor environments, HAR may play a crucial role in assisting the process of indoor localization and navigation. For example, if a user’s reported activity is going upstairs or taking an elevator upwards, one may infer an approximate location of the user to be near a staircase or an elevator, respectively. Having this in mind, and during the course of this work, we focused on the activities which may contain and provide context information for indoor positioning and navigation. Therefore, nine different activities were selected for classification: not moving, walking, running, riding an elevator up, riding an elevator down, walking upstairs, walking downstairs, or going up and down a ramp. All activities are described in Table 1.

All activities’ data were recorded continuously over time and therefore encompassed also the transitional periods/moments between different activities. For instance, the subject started the acquisition with the *not moving* activity and then switched to the *walking* activity in the moment that they started to walk naturally; in this case, both activities were annotated within the entire recording. This annotation process was used for the entire data-collection process, taking into account the different performed activities. By using this approach, the annotation of all the transitional moments between different and adjacent activities could be included in the dataset. It is also worth mentioning the particular method used when annotating elevator activities. In terms of annotation, the *elevator* activity begins at the moment that the elevator doors start to close, with the user already inside the elevator, and ends by the moment that the elevator doors start to open again, with the elevator already stopped at the destination floor. Moreover, the use of this specific annotation process for the *elevator* activity case is of utmost importance, especially for the evaluation and discussion of the model’s performance (described in detail in Section 6).

#### 5.1.2. HAR Dataset

For HAR model development, a dataset with the nine activities described in Section 5.1.1 was gathered. The main purpose of this dataset was to train and evaluate the performance of the proposed HAR model before its integration in the indoor positioning system. This dataset was collected using more than 15 different smartphones (low, mid and high-range smartphones) from different manufacturers (Samsung, Google, Huawei, Oneplus, Asus, Motorola, LG, Xiaomi) and Android versions (Android 5–10). For the smartphone usage, positions where the user can be actively using the smartphone for the purpose of positioning itself were selected. These positions include both texting and calling usages due to their common usage in an indoor navigation scenario. Scenarios where the smartphone is not being used, such as lying on a table or in a pocket, where not considered for acquisition since these positions imply that the application is running in background. Data from three-axis accelerometer, gyroscope, and magnetometer sensors were recorded with a sampling frequency ranging from 50 Hz to 200 Hz, depending on the used smartphone, during an entire acquisition.

All data were collected using an in-house Android application that recorded smartphone sensor data and allowed event annotation by the registry of timestamps associated with a predefined activity. This application displayed on the screen all the predefined HAR activities as buttons, allowing the real-time annotation of every activity and transition simply by tapping the corresponding activity on the screen whenever the user switched their current activity.

Moreover, this dataset contained human activity-related data from 77 different users, collected in more than 8 different locations/buildings. Part of this dataset consisted of retrospective data, which were acquired over the years from 2013 until 2020 for traditional human activity recognition tasks such as walking, standing, sitting, and going downstairs or upstairs.

For the training set, all retrospective data and a set of newer data were used, resulting into a total of approximately 21 h of useful data. Regarding test set, three different subjects collected data in new locations and buildings to fully assess the overall robustness and generalization capability of the trained model. The test set had a total of approximately 41 min of data. The number of samples per activity for each dataset is presented in Table 2.

#### 5.1.3. Indoor Location Dataset

To study the applicability of HAR within an IPS, an indoor location dataset was collected. Data were collected using the in-house Android application with annotation capabilities, which opportunistically recorded the sensed data. A set of predefined routes, with a predefined number of checkpoints, was previously designed for an office building. Therefore, this dataset was composed of inertial data (accelerometer, gyroscope), environment data (magnetometer, barometer, Wi-Fi, blueprint), and ground truth information about the localization in 3D coordinates (x, y, z) of route checkpoints. The annotation of the walked paths was conducted while the user was walking through the building at their normal speed. For each designed route, a predefined number of checkpoints was used to annotate the time instant when a checkpoint was reached. This annotation was conducted by the registry of timestamps when a user tapped the screen. Regarding the annotation of activities, each designed route included a checkpoint before and after a floor transition. For instance, when the user was going upstairs, the user needed to tap the screen before starting to climb the stairs and when the ascent ended. With this information, it was possible to have ground truth information about localization and activities.

**Figure 6:**
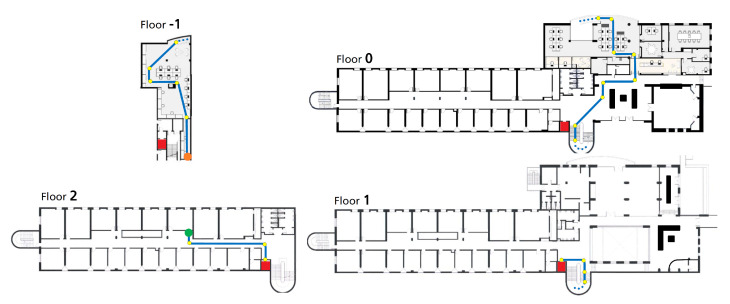
Office building floor plans with an example of a designed test route. The green circle represents the starting point, and the orange circle shows the last point. The yellow points are the checkpoint positions used for ground truth annotations. The red squares are the positions of the elevators, and the dotted line is a transition through a staircase.

This data-collection process took place in an office building with four floors, where the transitions between floors could be made by elevators or stairs. For a total accessible area of 1315 m^2^, divided by four floors, three different users collected data over 25 predefined trajectories, totaling 29 acquisitions. In terms of transitions, 20 and 17 transitions in total were recorded that were either performed using elevators or stairs, respectively. Three smartphones were used during the acquisitions, which were held in a texting position. In Figure 6, an example of a designed test route that goes through the four floors of the building is represented. This test route has 23 checkpoints, with two transitions made by stairs and one by elevator.

### 5.2. HAR Classification Performance

**Figure 7:**
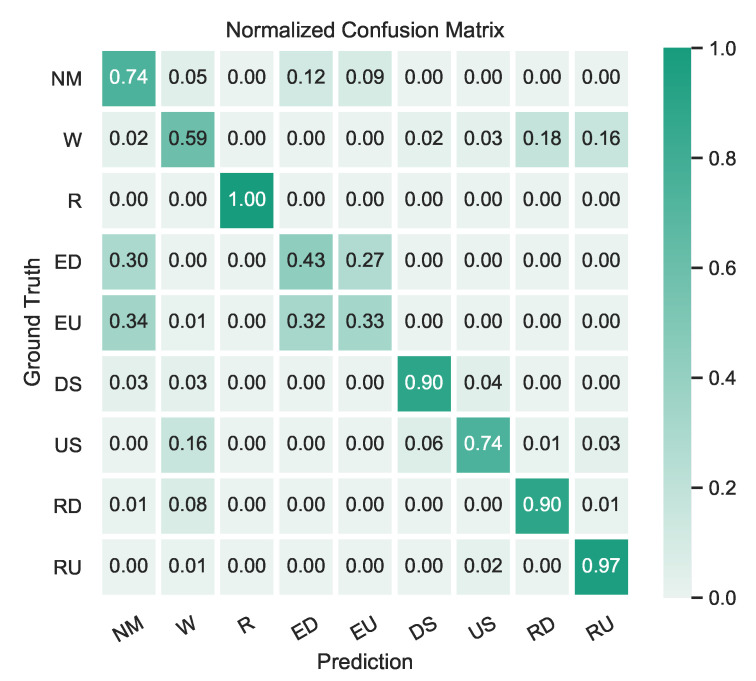
Normalized confusion matrix for test dataset.

The classification performance for each individual HAR activity is presented in this section. Figure 7 displays the classification performance attained by the model for the identification of each proposed human activity for the test dataset.

**Figure 8:**
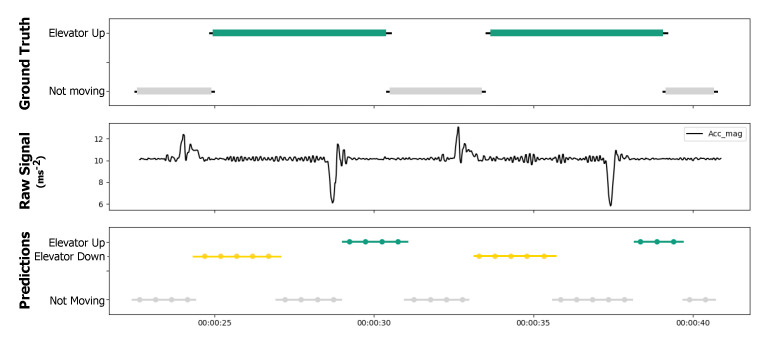
Illustrative example of HAR model predictions for an elevator up activity: ground truth annotations (**top**), accelerometer magnitude signal (**middle**), and predicted activities (**bottom**). For each presented elevator ride in the ground truth annotations, both “not moving” and “elevator up/down” activities are predicted. An “elevator” activity is usually predicted after observing a peak in the acceleration signal caused by the elevator initial and end movements, while the “not moving” activity is predicted for the remaining time where the user is standing still in the elevator (no significant changes in the acceleration signal).

As can be seen in Figure 7, the proposed method achieved an overall classification accuracy of 73% when detecting nine different human activities. Overall, the proposed method provided quite reasonable and accurate results for the classification of the proposed activities using only a smartphone’s inertial sensor data. However, the provided results showed that there is a small difficulty for the proposed model to distinguish between *walking* (W) and *ramp up/down* (RD-RU) activities, as well as when determining the direction of the movement (e.g., going up or going down). A more challenging discrimination occurs between *not moving* (NM) and *elevator up/down* (ED-EU) activities. Despite the low performance in identifying *elevator* (ED-EU) activities, it is important to note that the annotation process was made during the entire elevator ride, as stated in Section 5.1.1. Since an elevator ride includes instants of *not moving* (A0), these results are comprehensive and expected. An illustrative example of the latter scenario is presented in Figure 8. A more detailed discussion is presented in Section 6.

### 5.3. Indoor Localization Performance

The performance of the integration of the HAR model in the IPS is presented in this section. By default, this solution uses pressure data acquired by the barometer sensor to detect a transition between floors. However, the barometer sensor is not always presented in common smartphones, which reduces the scalability of this approach. Therefore, one of the contributions of this paper is to demonstrate the feasibility of the proposed integration of the HAR model in an IPS. For this purpose, the proposed IPS solution was evaluated using the indoor location dataset, in the presence and in the absence of the HAR model and barometer sensor, to detect a floor transition.

Table 3 presents the results obtained by the IPS solution for the indoor positioning scenario by testing the system using three approaches for detecting a floor transition: (1) using the pressure data acquired by the barometer sensor; (2) using the predicted HAR of the proposed model; and (3) using a hybrid approach where both barometer and HAR predictions are combined.

To enable the system evaluation, the IPS could check some positions of the designed trajectory annotated by the user during the acquisition. These expected locations were compared with the positions achieved by the system given by the centroid of all particles at each step. The closer each centroid was to the expected location, the higher the system performance. Thus, the IPS results were presented through the average centroid error, the percentage of correctly performed floor transitions, and the final positional error of the particle with the highest probability. Each tested acquisition was evaluated 10 times by the IPS solution to accommodate the random factor in particle distribution.

Overall, the average indoor localization error did not exceed 3.4 m when considering the average centroid error of all routes. Additionally, a lower error could be observed when the HAR module was incorporated within the IPS solution, reaching 2.41 m. Moreover, the proposed solution was also able to correctly identify and perform a floor transition in more than 84% of the cases, which can be crucial for use in multi-story buildings.

## 6. Discussion

The results obtained with the proposed method demonstrate its suitability to identify and classify several different human activities. Moreover, besides the traditional activities, such as moving or walking, more complex activities, such as taking an elevator up/downwards or walking up/down a ramp, were also accurately identified. A considerable number of data were used in this study, especially in the training dataset, and the collected data were quite diverse in their nature (with data coming from 77 different users) and in terms of locations (different buildings and environments), ensuring that data variability was present both in training and test data. Having this variability present in the data is an excellent way to effectively evaluate the model performance for the recognition of a broad range of activities within different scenarios.

In terms of HAR classification results, a minimum overall accuracy of 73% was obtained when detecting nine different activities. However, looking directly at this presented value can be misleading, if the overall scenario is not taken into account. Thus, it is worthwhile to mention and discuss some of the model’s erratic behavior when predicting certain types of activities. First, it can be seen that the model sometimes confused walking with going up/down a ramp. This behavior was expected as, roughly speaking, the main difference between walking normally on a flat surface or walking up/down a ramp is that the ground’s inclination/slope changes. These results may suggest that the model is more prone to classify a ramp up/down activity whenever the ground is not perfectly leveled, or a minimum amount of inclination is present. Moreover, since by design no pressure data are used in the proposed model, there is no direct means for the model to accurately estimate the slope change in the input data at this finer scale and for these activities. Nevertheless, this type of situation only occurs in a small portion of the evaluated data, and thus does not compromise the overall results of the proposed method or IPS solution.

Secondly, it is also worth mentioning the more frequent confusion between the not moving and elevator up/down activities. In this case, it is quite common for the model to misclassify a given time segment within an elevator ride, as with the not moving activity rather than the elevator up/down activity. Once again, by taking into account how an elevator ride was annotated—i.e., it began when the elevator door started to close and ended when the elevator door started to open—and the fact that there was no direct way to accurately estimate altitude using the input sensor data, the authors find this behavior normal or at least expected. Specifically, an elevator ride usually consists of walking into the elevator, pressing a button, standing still during the ascent/descent of the elevator and finally exiting, which indeed may explain the model’s confusion when it predicts not moving as the predominant activity for a great part of the elevator ride. Moreover, and after performing a visual inspection of some of these cases (see Figure 8), it can be concluded that the model may have learned to identify and characterize the elevator ride mostly based on the initial and end elevator accelerations, with the remaining parts of the ride generally classified as not moving activity, which is, in fact, also correct.

Lastly, and still regarding the elevator activity, the proposed model tended not to be able to fully identify the correct direction of the elevator ride—i.e., if it was going up or down—as it classified more down than up directions for most elevator samples. However, if we choose to ignore the provided elevator ride direction in the model’s prediction, an overall accuracy of approximately 67% was obtained for the elevator activity identification using only inertial sensor data. Moreover, the use of a barometer sensor could help in solving and dealing with the aforementioned situations as suggested and highlighted in the work of Zhou et al. [4]. They attained a classification accuracy for the elevator activity of 98% by also including barometer data into their model, attaining substantially higher results compared to our model (67%) which did not use barometer data. However, it was not considered by design, as the goal of this study was to develop a model that did not require the use of the barometer sensor, in order for the proposed solution to be used in even more smartphones (a barometer is not present in most low- to mid-range smartphones) and thus to reach more end users in real-life scenarios.

Regarding HAR, the proposed model effectively captured the intrinsic characteristics and dynamics of each underlying activity under different settings and with demonstrated results. It is also worth mentioning that the model was evaluated using test data that were collected in new locations and environments—i.e., different elevators, stairs or ramps that were never used by the model until the evaluation moment—while still demonstrating adequate and accurate results. These results are in line with those ones reported in the literature [4], which shared the goal of extracting semantic information from the human activities which then can be used for indoor localization. Still, no direct comparison can be made since the predicted activities differ in number and/or complexity, the input data are not the same (e.g., inertial vs combination of inertial and pressure data), the annotation process is different and/or the dataset is not publicly available for comparison.

In terms of indoor location, the inclusion of the HAR model output demonstrated the ability to improve the obtained results compared to the proposed solution baseline. By effectively tracking the user’s current activity, it was possible to reduce uncertainty about the indoor positioning of the user and to detect floor transitions more accurately, improving the overall robustness of the solution. As in Table 3, even without resorting to the use of barometer data in the IPS, 84% of the floor transitions were still detected using the HAR module against 66% where no HAR information was available (where only pressure data could indicate floor changing). The latter behavior may be related to the fact that the proposed system verifies each generated particle position frequently by crossing that information with the corresponding floor structure and by verifying if a given position is or is not possible. By using HAR, we could determine, for instance, if a user was currently going upstairs or taking an elevator, which increased the system’s confidence in those particles that were currently on or near stairs or elevator locations, assisting in the process of identifying the user’s relative location within a building, especially during the floor transitioning process. Thus, an accurate tracking of user’s activities indoors could actively contribute to the improvement of the given IPS, which works under these assumptions.

One of the goals of this study was to incorporate HAR into an IPS in a way that the system could even operate without the use of barometer data, which was mandatory in our solution, by exclusively relying on HAR to detect floor transitioning. Even though atmospheric pressure data can undoubtedly positively contribute to an increase in overall performance resulting in smaller positional errors (a decrease of 0.11 m), with the higher number of detected floor transitions (an increase of 5%), it can now be considered not to be fundamental for the whole system to work. This fact is of utmost importance as, by extending the use of the proposed IPS solution to devices that do not contain the barometer sensor, we could provide our solution to more users, by reaching more smartphones, especially low- to mid-range devices, and provide a means of localization without having to use barometer data. Nevertheless, the conducted study has its limitations, as the used IPS dataset does not contain any floor transition made by a ramp, and the method should therefore be subject to further validation, with a larger dataset and subsequently in a real-world scenario.

## 7. Conclusions

In this paper, we proposed an activity recognition model for detecting human activities in the context of indoor positioning. A deep-learning model based on ConvLSTM layers was developed for the classification of nine different human activities based on accelerometer, gyroscope, and magnetometer smartphone sensor measurements. This model is capable of not only correctly classifying simpler activities, such as walking or not moving, but also more complex activities, such as taking an elevator, ascending/descending stairs, or walking up/down a ramp. The proposed HAR model was evaluated in a complex and independent test dataset with accurate results, demonstrating that the proposed model effectively captured the intrinsic characteristics and dynamics of each underlying activity and under different settings.

A novel method for incorporating semantic information extracted from the performed human activities within the proposed indoor positioning system was also presented, improving current IPS results. An average positioning error of 2.4 m was obtained using the proposed IPS in combination with HAR on 29 predefined testing routes. Moreover, more than 84% of floor transitions were correctly identified and performed in those routes, contributing to a better estimation of the user’s current position in a multi-story building without using any reference to atmospheric pressure data.

Future work will include experimentation on a larger-scale dataset and exploring transfer learning techniques to extend the number of monitored activities, as well as exploring more efficient ways to incorporate and integrate human activity-related semantic information within the proposed IPS.

## Figures and Tables

**Table 1:** Proposed human activities for classification.

ID	Activity	Description
NW	Not Moving	The user is seated, standing, or waving their smartphone around without actually moving.
W	Walking	The user is walking naturally.
R	Running	The user is running.
ED	Elevator Down	The user is taking an elevator downward (one or more floors).
EU	Elevator Up	The user is taking an elevator upward (one or more floors).
DS	Down Stairs	The user is going downstairs.
US	Up Stairs	The user is going upstairs.
RD	Ramp Down	The user is going down a ramp.
RU	Ramp Up	The user is going up a ramp.

**Table 2:** Training and test dataset sample distribution.

Activity ID	Number of Samples	
	Train	Test
NM	18,590	1147
W	16,980	1512
R	605	76
ED	4569	436
EU	4639	434
DS	7285	170
US	8402	237
RD	7892	355
RU	8471	340

**Table 3:** IPS performance results for a total of 29 evaluated routes (indoor location dataset).

	Barometer	HAR	Bar + HAR
**Average Centroid Error (m)**	3.33 ± 0.07	2.52 ± 0.12	**2.41** ± **0.06**
**Correct Floor Changes (%)**	66 ± 1	84 ± 1	**89** ± **1**
**Best Particle Final Positional Error (m)**	6.19 ± 0.57	3.73 ± 1.10	2.84 ± 0.83

## Data Availability

The data presented in this study are available on request from the corresponding author. The data are not publicly available since official permission is required.
